# Patient-Controlled Intravenous Analgesia with or without Ultrasound-Guided Bilateral Intercostal Nerve Blocks in Children Undergoing the Nuss Procedure: A Randomized, Double-Blinded, Controlled Trial

**DOI:** 10.1155/2022/5776833

**Published:** 2022-07-22

**Authors:** Bingjie Ma, Yuan Sun, Can Hao, Xiaoming Liu, Sai'e Shen

**Affiliations:** ^1^Department of Pain Management, Xinhua Hospital Affiliated to Shanghai Jiaotong University School of Medicine, Shanghai, China; ^2^Department of Anesthesiology and Intensive Care Medicine, Xinhua Hospital Affiliated to Shanghai Jiaotong University School of Medicine, Shanghai, China

## Abstract

**Background:**

Two analgesic strategies have been described for pain treatment after the pectus excavatum surgery: the patient-controlled intravenous analgesia (PCIA) and ultrasound-guided intercostal nerve block. In this prospective, randomized and double-blinded trial and the short and long-term outcomes were compared in patients after surgery.

**Methods:**

The children were randomized to either the intercostal or control group. Ultrasound-guided intercostal nerve block was with 0.25% ropivacaine and 5 mg dexamethasone in the intercostal group, while the control group was with 0.9% normal saline. The block was performed in the intercostal space corresponding to the lowest depression of the sternum and repeated bilaterally in the spaces above and below. Postoperatively, the children in the two-groups received PCIA with fentanyl for 48 hours. The primary outcome was a pain score on the postoperative day 1, as measured by the Visual Analogue Scale (VAS).

**Results:**

Sixty children undergoing the Nuss procedure were enrolled in the trial. The mean differences in VAS scores between the two groups were 3.2 in the PACU (*p* < 0.001), 1.7 on postoperative day 1 (*p* < 0.001) and 0.7 on postoperative day 2 (*p*=0.015). The opioid consumption was significantly lower in the intercostal group during the postoperative 48 hours (*p* < 0.05). The anxiety and QOL scores in the intercostal group were significantly improved on some points of time (*p* < 0.05). The incidence of adverse events was markedly lower in the intercostal group during the postoperative 48 hours (*p* < 0.05).

**Conclusions:**

Our results suggest ultrasound-guided intercostal nerve block with PCIA may be more effective than PCIA alone in children who underwent the Nuss procedure.

## 1. Introduction 

Pectus excavatum is a common congenital chest wall deformity that occurs in 0.1% of live births [1]. The Nuss procedure is a minimally invasive approach for this abnormality, but the postoperative pain is severe and is considered a significant problem [2].

Many analgesic strategies have been described to manage postoperative pain after the Nuss procedure, such as patient-controlled intravenous analgesia (PCIA), multimodal analgesia, thoracic epidural block, paravertebral block, and intercostal block. Epidural analgesia is established as a safe and effective method for postoperative pain management in children. However, the risks of thoracic epidural analgesia challenge its superiority in pain treatment. They are infection, nerve damage, drug error, and local anesthetic toxicity [3]. Unlike lumbar epidural block, thoracic epidural block is associated with a higher risk of neurological complications due to the presence of the spinal cord close to the place where the needle and catheter are inserted [4]. Finally, there is a discrete failure rate in the placement of thoracic epidural block, which ranges from 5% to 30% [5].

PCIA is a well-established method for relieving postoperative pain in children and adolescents [6], which is as effective as thoracic epidural block in decreasing postoperative pain after the Nuss procedure with fewer complications [7]. However, PCIA does have minor and major adverse effects, such as nausea, vomiting, urinary retention, sedation, or respiratory depression [8].

An intercostal nerve block is considered less invasive than epidural blockade and is relatively simple to perform and successful in children who have undergone thoracic and upper abdominal procedures [9]. The advantages of intercostal nerve block include good analgesia, opioid-sparing effect, improved pulmonary mechanics, reduced central nervous system depression, and absence of urinary retention [10]. Ultrasound-guided intercostal nerve block can improve the effectiveness and safety of the intercostal nerve block. Moreover, ultrasound-guided intercostal blocks could be an interesting alternative when an epidural or paravertebral block is difficult or impossible to perform such as in the case of severe scoliosis or vertebral anomalies.

A retrospective cohort study of adolescents and young adults who underwent the Nuss procedure showed that some of them suffered from chronic postsurgical pain [11]. In our clinical practice, we also observed that some children still complained about pain six months postoperatively. We previously demonstrated that ultrasound-guided intercostal nerve block was more effective than PCIA for postoperative acute pain in children after the Nuss procedure [12]. Therefore, this study aimed to explore whether the addition of dexamethasone to ultrasound-guided intercostal nerve block with PCIA (intercostal group) would have better analgesic (acute and chronic) or other positive effects than PCIA alone (control group). The primary outcome was pain scores on postoperative day 1, as measured by the Visual Analog Scale (VAS).

## 2. Materials and Methods

### 2.1. Participants

**Figure 1:**
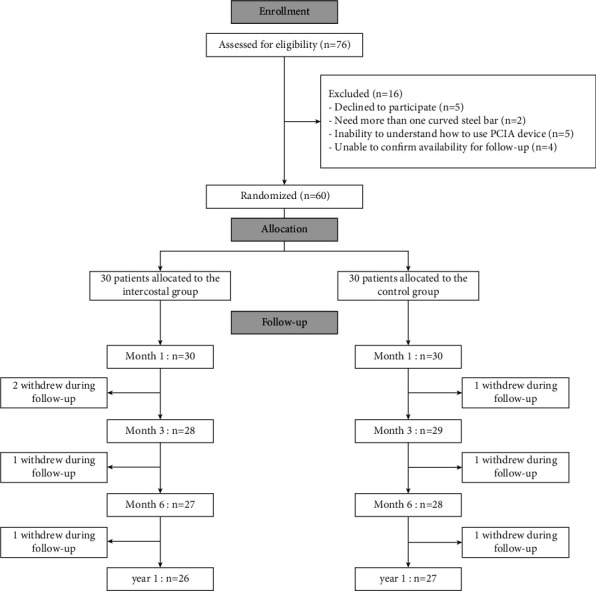
Participant flow diagram for the study. PCIA, patient-controlled intravenous analgesia.

Patients with pectus excavatum were enrolled between September 1, 2018, and August 31, 2019. This study was approved by the ethics committee (approval no.: XHEC-C-2018-018–2) and prospectively registered at http://www.chictr.org (ChiCTR1800018040). The exclusion criteria were as follows: patients requiring additional surgery, history of analgesic administration (such as opioids, acetaminophen, or nonsteroidal anti-inflammatory drugs) 24 hours before premedication, history of coagulation disorders or allergic to local anesthetics, history of renal insufficiency or American Society of Anesthesiologists (ASA) physical status of higher than II, inability to understand PCIA device use, or parental objection to intercostal nerve block. The day before surgery, all children and their parents were informed about the trial process and signed inform consent in the anesthetic assessment office. The children (age range, 10 to 16 years) scheduled for the Nuss procedure at Xinhua Hospital were randomized to the intercostal or control group (Figure 1).

### 2.2. Study Protocol

All children were premedicated with midazolam 0.3 mg/kg and acetaminophen 15 mg/kg (maximum 650 mg) orally. After obtaining intravenous access, all subjects were anesthetized with intravenous midazolam 0.1 mg/kg, propofol 3 mg/kg, fentanyl 2 *μ*g/kg, and rocuronium 0.6 mg/kg. After that, the trachea was intubated with an appropriately sized tracheal tube (Covidien llc, single lumen, with cuff), and sevoflurane (end-tidal concentration, 2%) in nitrous oxide (fraction of inspired oxygen, 0.5) was administered as an anesthetic. Remifentanil (0.2 *μ*g/kg/min) was continuously injected intravenously as an intraoperative analgesic. Patients received intravenous ondansetron hydrochloride 0.1 mg/kg before recovery. In this study, ropivacaine was used because it has less motor blockade effects and less cardiac toxicity than bupivacaine (high absorption of local anesthetic injected in the intercostal space). However, ropivacaine alone is associated with a shorter duration when used for nerve block. Dexamethasone when combined with ropivacaine can prolong the nerve block time of local anesthetics [13]. Therefore, in this study, dexamethasone 5 mg was added to the ropivacaine solution.

**Figure 2:**
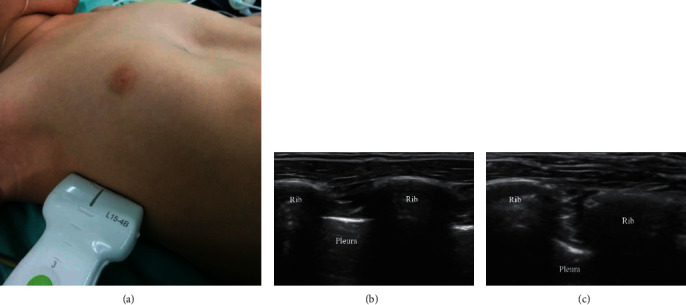
Ultrasound image of intercostal nerve blocks. (a) Placement of the ultrasound-probe for the performance of intercostal nerve block. (b) The hyperechoic ribs and the pleura. (c) After the administration of the local anesthetic, the bolus increased, and expansion of the potential space is visible.

After intubation, the patient was placed in supine position, and an ultrasound-probe (M-Turbo® with L25 transducer, SonoSite® Inc.) was used to scan the thoracic wall laterally from the midaxillary line and identify the required anatomic landmarks. The ribs were identified as hyperechoic streaks, while the pleura appeared as hyperechoic lines between and below the ribs. The anesthetist used a 22-G short-bevel needle to introduce 0.25% ropivacaine hydrochloride (Table 1) and 5 mg dexamethasone in the intercostal group or normal saline solution (0.9%) in the control group at the incision site. The needle tip was advanced under ultrasound guidance until the surface of the pleura and the spread of the injected solution (local anesthetic or Nsaline) were visualized (Figure 2). This procedure was carried out in the intercostal space corresponding to the lowest depression of the sternum and repeated bilaterally one space above and one space below. Accordingly, a total of 6 injections were given to each patient.

Postoperatively, the children in the two-groups received PCIA with fentanyl for 48 hours. The PCIA settings were a basal infusion of 0.5 *μ*g/kg/h and a 0.25 *μ*g/kg bolus dose with a 30-minute lockout period. All children were also given NSAIDs orally.

### 2.3. Postoperative Follow-Up

Postoperatively, all participants were transferred to the postanesthesia care unit (PACU). When the children regained consciousness, a blinded research team member assessed their postoperative pain levels by using VAS in both groups. The additional VAS pain scores were assessed on preoperative day 1 (baseline) and postoperative day 1, day 2, day 7, month 1, month 3, month 6, and year 1. If the pain score was ≥4, fentanyl 0.2 *μ*g/kg was administered as a rescue analgesic. PCIA press number, rescue analgesics, total fentanyl consumption, and analgesia-associated side effects, including respiratory depression (defined as a respiratory rate <8 bpm), requirement of naloxone, and/or peripheral oxygen saturation <90% as evaluated by an author, urinary retention, pruritus, nausea, and vomiting were recorded during the first 48 hours after surgery. The anxiety scores, depression scores, and quality of life (QOL) were measured preoperatively and on postoperative day 7, month 1, month 3, month 6, and year 1. After discharge, the children would be followed up at the outpatient clinic 6 months later, while other times were by telephone or WeChat.

### 2.4. Blinding

Using the sealed envelope technique, the patients were randomized into one of the two groups: the control group and the intercostal group. The two anesthetic techniques were explained to all patients before they were randomly allocated to one of the groups. An anesthesiologist took care of randomization and prepared all local anesthetic solutions, drugs, and PCIA pumps. Other anesthesiologists, patients, medical staff members, and the statisticians were unaware of the treatment groups.

### 2.5. Outcomes

The primary outcome included pain scores on postoperative day 1, as measured by VAS. Children assessed their pain level using an 11-number scale varying from 0, which indicated no pain, to 10, which indicated unbearable pain. The pain scores were noted every 6 hours, and we averaged each 24-hour period postoperatively. The secondary outcomes included pain-related, mental health, QOL, and safety. Pain-related outcomes included VAS scores at other time points, PCIA press number, rescue analgesics, and total fentanyl consumption at 48 hours postoperation. Mental health included anxiety and depression at every time point during the first postoperative year. Safety-related outcomes included assessment of adverse effects. In the study, the screen for Child Anxiety-Related Emotional Disorders (SCARED) was used to evaluate anxiety, Children's Depression Inventory (CDI) was used to assess depression, and Pediatric Quality of Life Inventory version 4.0 Generic Core Scales (PedsQL4.0) was used to value QOL. The higher the SCARED score, the higher the anxiety and a total score ≥23 is considered to indicate the presence of anxiety. CDI is the most widely used self-rating scale for children aged 7–17 years. A total score ≥20 is considered as indicating the presence of a depressive disorder, and the higher the score, the more severe the depression [14]. The PedsQL 4.0 is widely used for children aged 2–18 years: out of 100, the higher the score, the better the quality of life [15].

### 2.6. Sample Size Calculation

PASS (version 15) was used to calculate the required sample size based on the pain scores on postoperative day 1 as the primary outcome variable. The result of preliminary study showed that the difference value of pain on postoperative day 1 was 1.4 between the two groups, the standard deviation for the intercostal group was 1.64, while the same in the control group was 1.73. The power of test 1 − *β* was 0.8. Using the one-sided value of *α* = 0.025, the sample size of each group should be at least 24. Accordingly, 60 children were enrolled, while the dropout rate was 20%.

### 2.7. Statistics

SPSS (version 22) was used for conducting statistical analysis. The data collected contained both continuous and categorical variables. The normality of the data was evaluated using the Kolmogorov–Smirnov test. Data with normal distribution were summarized as means and SD, while data with nonnormal distributions were summarized as medians and IQR. Repeated measures ANOVA test was conducted to compare the changes from baseline in the VAS pain scores at PACU and postoperative day 1, day 2, day 7, month 1, month 3, month 6, and year 1. The same statistics were used for anxiety, depression, and QOL. Mann–Whitney *U*-test was used for comparing the PCIA press number, rescue analgesics, and total fentanyl consumption. For data with normal distribution, we calculated the mean differences in scores between the two groups and their 95% confidence interval (CI), while we obtained median differences and 95% CI for data with nonnormal distribution. Age, body mass index, and duration of surgery were compared using 2-tailed *t*-tests, while the incidence of analgesia-associated side effects was compared using 2-tailed Fisher's exact test. A 2-sided *P* value of <0.05 was considered statistically significant. Data are represented as either ratio (percentage) or mean (SD) or median (IQR).

## 3. Results

Of the 76 enrolled patients, 16 were excluded due to the requirement of additional surgery (more than 1 curved steel bar, *n* = 2), refusal to participate (*n* = 5), inability to understand how to use the PCIA device (*n* = 5), or inability to confirm availability for follow-up (*n* = 4). Eventually, 60 patients were successfully randomized to assign to the intercostal (*n* = 30) or control group (*n* = 30; Figure 1).

The patients in the two study groups were similar in age, sex, body mass index, ASA classification, and operation duration (Table 2). A total of 6 ultrasound-guided intercostal injections were performed in each patient: the intercostal spaces were bilaterally T4–6(80%), T3–5(20%). No complications associated with the use of ultrasound-guided intercostal nerve block, such as pneumothorax, pleural puncture, penetration of the peritoneum and abdominal viscera, nerve injury, and intravascular injection were observed.

**Figure 3:**
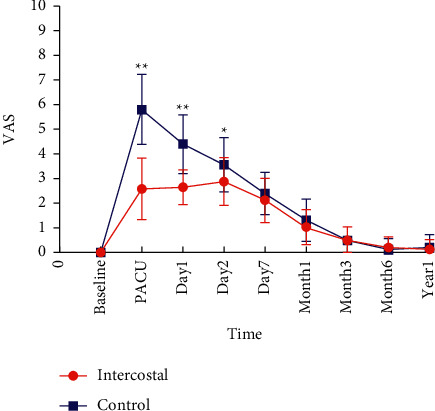
VAS scores after surgery. ^∗∗^*P* < 0.01 and ^*∗*^*P* < 0.05. VAS, Visual Analog Score; PACU, postanesthesia care unit.

Our primary outcome was VAS scores on postoperative day 1. The mean difference in the VAS score between the control and intercostal group was 3.2 (*p* < 0.001) in the PACU, 1.7 (*p* < 0.001) on postoperative day 1, and 0.7 (*p*=0.015) on postoperative day 2 (Table 3). VAS scores were similar on the other days (Table 3). The postoperative pain scores of the two groups returned to the preoperative level on postoperative month 6 (Figure 3). The PCIA press number, the rescue analgesics consumption, and opioid consumption were significantly lower in the intercostal group when compared with the control group in the PACU, on postoperative day 1 and day 2 (*p* < 0.05, Table 3).

**Figure 4:**
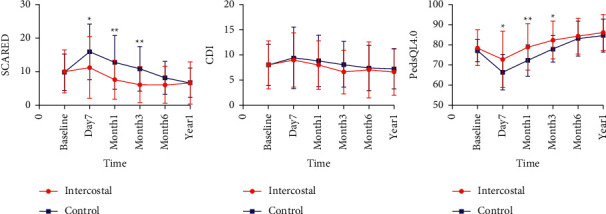
Anxiety scores (SCARED), depression scores (CDI), and quality of life scores (PedsQL4.0) after surgery. ^∗∗^*P* < 0.01 and ^*∗*^*P* < 0.05. SCARED, Screen for Child Anxiety-Related Emotional Disorders; CDI, Children's Depression Inventory; PedsQL 4.0, Pediatric Quality of Life Inventory version 4.0 Generic Core Scales.

SCARED scores were significantly lower in the intercostal group than the control group postoperatively (POD7: 11.3 ± 9.2 vs. 15.9 ± 8.3; POM1: 7.6 ± 5.8 vs. 12.8 ± 8.0; POM3: 6.1 ± 5.3 vs. 10.9 ± 6.6, *p* < 0.05) (Table 4). In the intercostal group, the SCARED scores improved than baseline from postoperative month 1, while the SCARED scores improved on postoperative year 1 in the control group (Figure 4). CDI scores showed no significant differences between the 2 groups. The PedsQL4.0 scores were higher in the intercostal group than the control group on postoperative day 7, month 1, and month 3 (POD7: 72.8 ± 14.1 vs. 66.4 ± 8.7; POM1: 79.0 ± 11.5 vs. 72.3 ± 7.8; POM3: 82.3 ± 9.4 vs. 78.0 ± 6.7, *p* < 0.05) (Table 4). The PedsQL4.0 scores showed improvement from postoperative month 6 in both groups (Figure 4).

The incidence of adverse events was markedly lower in the intercostal group in the PACU (3.3% vs. 26.7%, *p*=0.03) and in the postoperative 48 hours (23.3% vs. 60%, *p*=0.004) than the control group (Table 5).

## 4. Discussion

The results of this study showed that ultrasound-guided intercostal nerve block with the addition of dexamethasone for pain management caused by the Nuss procedure decreased the VAS scores and opioid consumption during the postoperative 48 hours. The patients in the intercostal group also demonstrated lower anxiety scores from postoperative day 7 to postoperative month 6 and better QOL scores from postoperative day 7 to postoperative month 3.

In the current study, the ultrasound-guided intercostal nerve block provided an important opioid-sparing effect and superior postoperative analgesia during the first 48 h after surgery. This is partly consistent with the results obtained from our previous study, wherein the postoperative FPS-R scores were reduced only for the first 6 hours [12]. This might be partly due to the effects of the dexamethasone added to ropivacaine. Previous studies have indeed shown that adding dexamethasone enhances the effects of peripheral nerve block and prolongs the duration of the sensory block of local anesthetics used for peripheral nerve blocks [16, 17]. Possible beneficial mechanisms of dexamethasone include peripheral/central anti-inflammatory effects, inhibition of ectopic neural discharge, suppression of nociceptive C fibers, and suppression of the neuropeptide immune response to injury [18]. Lukosiene et al. have found that VAS scores of pain at rest were significantly lower for up to 3 h after surgery in a single shot bilateral intercostal block with levobupivacaine, but no differences were observed 6 h after surgery [19]. The success rates associated with conventional approaches are highly dependent on operator skills and are associated with potential serious complications. In our study, the bilateral intercostal block and anesthetic agent spread was monitored by ultrasonography, which provides real-time information about the needle tip location and local anesthetic delivery to the desired location [20]. Previous studies have demonstrated that ultrasound-guided intercostal nerve block could decrease postoperative pain intensity in patients undergoing chest and upper abdominal surgery [21].

Our study not only investigated the acute pain after pectus excavatum surgery but also the postoperative chronic pain. No significant difference was observed between the two groups, the probable reason may be that our study was not powered enough to study this outcome, and some methodological issues are of concern (see limitations). And, after postoperative 3 months, the postoperative pain basically disappeared, and only a slight pain was observed in a few patients. To the best of our knowledge, most of the studies have focused on acute pain after pectus excavatum surgery, and this is the first study to follow-up postoperative pain for one year.

Pectus excavatum is the most common congenital chest wall deformity, but little is known about the influence of the Nuss surgical procedure on the mental health of patients. In each group, surgery could help improve the mental health of patients, which was consistent with that of the previous study [22, 23]. The anxiety scores and QOL scores in the intercostal group were significantly improved than the control group on some points. Intercostal nerve block could reduce the stress response in patients after surgery. Zhan et al. have reported that the intercostal nerve block in patients undergoing minimally invasive mitral valve surgery inhibits the stress response to surgery by reducing the levels of cortisol, glucose, IL-6, and TNF-*α* [24]. The stress response might lead to changes in the nervous, endocrine, and immune systems, causing alterations in the metabolic processes and functions. This might explain why the anxiety scores were lowered in the intercostal group. The PedsQL4.0 is composed of body domain, emotional function, social function, and school performance. In our study, the anxiety scores were significantly improved in the intercostal group than the control group postoperatively, so the QOL might be higher in the intercostal group accordingly.

The incidence of analgesia-associated adverse reactions was markedly lower in the intercostal group in during the first 48 postoperative hours, which may result from the difference in opioid consumption between two groups. Intercostal nerve block might cause adverse events, such as pneumothorax, peritoneal penetration, and abdominal viscera. However, under the guidance of ultrasound, its effectiveness and safety showed great improvement.

We believe one of the strengths of our study is the monitoring of the outcomes, which were assessed for one year after the surgery. A majority of the randomized controlled trials have followed up intercostal nerve block in patients for a short-term period, with recent studies following patients for 24 h [25], 48 h [26], or up to 1 week postoperatively [27]. By following patients for 1 year, we tried to study the change in postoperative pain, psychological state, and long-term effects of the two analgesic methods during the first postoperative year. Furthermore, 0.25% ropivacaine and dexamethasone 5 mg were used for intercostal nerve block in the present study. Additionally, in our study, ultrasound was used to guide the intercostal nerve block.

However, this study has some limitations. First, we did not take the educational level, psychological traits, and compliance of the patients when planning the follow-up phase of the study, and many children came from remote country villages. Second, we did not assess the severity of pectus excavatum, the occurrence of postoperative surgical complications, the time to return to normal activity, and the patient's satisfaction: all these items may also influence the child's mental state and the presence of postoperative pain. Third, our primary outcome was the pain scores on postoperative day 1, which were used to calculate the required sample size. Our study was thus probably not powered enough (not enough patients included) to evaluate differences in other items over a long period of time. Fourth, future studies using a liposomal local anesthetic with a prolonged effect could be performed to better evaluate its effect on acute and long-lasting postoperative pain. Finally, we only studied the children aged from 10–16 years old in the present study. In future studies, the age range of patients could be expanded to find possible difference among children of preschool, school-age, and adolescence.

In conclusion, our results demonstrated that the ultrasound-guided intercostal nerve block with adjuvant of dexamethasone might control acute pain and anxiety and improve QOL better. Our study suggests that ultrasound-guided intercostal nerve block with PCIA might be more effective than PCIA in children who underwent the Nuss procedure.

## Figures and Tables

**Table 1:** Total volume of local anesthetic.

Weight (kg)	Volume of single ropivacaine injection (ml)	Volume of total ropivacaine used
≥15–<30	3	18 ml (45 mg)
≥30–<45	4	24 ml (60 mg)
≥45	5	30 ml (75 mg)

**Table 2:** Demographic and baseline characteristics.

	Intercostal group	Control group	*P* value	Md 95% CI
Age in years	13.6 (1.9)	13.3 (1.8)	0.941	−0.3 (−1.2–0.7)
Male (%)	86.7	93.3	0.667	
Body mass index (kg/m^2^)	17.80 (2.5)	18.6 (2.3)	0.685	0.8 (−0.5–2.0)
*ASA class (%)*			0.121	
I	43.3	63.3		
II	56.7	36.7		
Duration of surgery (min)	41.8 (6.3)	45.5 (10.8)	0.257	3.7 (−0.9–8.3)

**Table 3:** Comparison of pain-related outcomes between the two groups.

VAS pain scores	Intercostal group	Control group	*P* value
Preoperative day 1	0	0	—
PACU	2.6 (1.3)	5.8 (1.4)	<0.001
Postoperative day 1	2.7 (0.7)	4.4 (1.2)	<0.001
Postoperative day 2	2.9 (1.0)	3.6 (1.1)	0.015
Postoperative day 7	2.1 (0.9)	2.4 (0.9)	0.234
Postoperative month 1	1.0 (0.7)	1.3 (0.8)	0.170
Postoperative month 3	0.5 (0.6)	0.5 (0.6)	0.901
Postoperative month 6	0.2 (0.4)	0.1 (0.4)	0.639
Postoperative year 1	0.1 (0.4)	0.2 (0.5)	0.519

PCIA press number			
PACU	0 (0–1)	1 (1–2)	<0.001
Postoperative 24 h	4 (2–5)	11 (9–12.3)	<0.001
Postoperative 24–48 h	3 (1–4)	7 (5–9)	<0.001
Postoperative 48 h	6 (5–9)	17.5 (13.8–20.3)	<0.001

Rescue analgesics (*μ*g/kg)			
PACU	0 (0–0.3)	0.9 (0–2.0)	0.020
Postoperative 24 h	0.1 (0–1.3)	2.7 (1.8–5.0)	<0.001
Postoperative 24–48 h	0 (0–1.0)	3.4 (2.1–4.0)	<0.001
Postoperative 48 h	0.2 (0–2.5)	6.1 (4.1–7.9)	<0.001

Total amount of fentanyl (*μ*g/kg)			
PACU	0.5 (0.5–1.0)	1.7 (0.8–2.6)	<0.001
Postoperative 24 h	13.3 (12.7–14.4)	17.1 (15.7–19.7)	<0.001
Postoperative 24–48 h	11.4 (11.0–11.9)	12.7 (11.7–13.3)	0.009
Postoperative 48 h	24.2 (24.0–26.5)	30.1 (28.1–31.9)	<0.001

**Table 4:** Comparison of psychological outcomes between the two groups.

	Intercostal group	Control group	*P* value
Anxiety scores (SCARED)			
Preoperative day 1	10.1 (6.4)	9.8 (5.4)	0.845
Postoperative day 7	11.3 (9.2)	15.9 (8.3)	0.044
Postoperative month 1	7.6 (5.8)	12.8 (8.0)	0.006
Postoperative month 3	6.1 (5.3)	10.9 (6.6)	0.003
Postoperative month 6	6.1 (5.5)	8.2 (4.9)	0.138
Postoperative year 1	6.7 (6.3)	6.7 (4.3)	0.973

Depression scores (CDI)			
Preoperative day 1	8.0 (4.8)	8.0 (4.1)	0.977
Postoperative day 7	9.0 (5.4)	9.4 (6.1)	0.769
Postoperative month 1	8.0 (4.8)	8.8 (5.1)	0.517
Postoperative month 3	6.6 (4.4)	8.1 (4.5)	0.197
Postoperative month 6	7.0 (5.6)	7.4 (4.5)	0.768
Postoperative year 1	6.6 (4.6)	7.3 (4.0)	0.530

QOL scores (PedsQL 4.0)			
Preoperative day 1	78.5 (9.0)	77.1 (5.5)	0.472
Postoperative day 7	72.8 (14.1)	66.4 (8.7)	0.04
Postoperative month 1	79.0 (11.5)	72.3 (7.8)	0.01
Postoperative month 3	82.3 (9.4)	78.0 (6.7)	0.041
Postoperative month 6	84.3 (8.9)	83.2 (8.6)	0.611
Postoperative year 1	86.1 (9.0)	84.7 (8.2)	0.515

**Table 5:** Analgesia-associated adverse events in the PACU and 2 days postoperatively.

	Intercostal group	Control group	*P* value
Analgesia-associated adverse events (PACU)			
Total	1 (3.3%)	8 (26.7%)	0.03
Nausea, vomiting	0 (0%)	1 (3.3%)	0.5
Pruritus	0 (0%)	0 (0%)	1
Urinary retention	0 (0%)	0 (0%)	1
Respiratory depression	1 (3.3%)	7 (23.3%)	0.058
RR <8 bpm	0 (0%)	2 (6.7%)	0.472
Naloxone administration	0 (0%)	0 (0%)	1
SpO_2_ <90%	1 (3.3%)	5 (16.7%)	0.197

Analgesia-associated adverse events (postoperative 48 hrs)			
Total	7 (23.3%)	18 (60%)	0.004
Nausea, vomiting	2 (6.7%)	7 (23.3%)	0.148
Pruritus	1 (3.3%)	1 (3.3%)	1
Urinary retention	0 (0%)	0(0%)	1
Respiratory depression	4 (13.3%)	10 (33.3%)	0.067

## Data Availability

The original data used to support the findings of this study are available from the corresponding author upon request.
